# Development of a new stroke scale in an emergency setting

**DOI:** 10.1186/s12883-016-0695-z

**Published:** 2016-09-08

**Authors:** Haifeng Mao, Peiyi Lin, Junrong Mo, Yunmei Li, Xiaohui Chen, Timothy H. Rainer, Huilin Jiang

**Affiliations:** 1 Emergency Department, The 2nd Affiliated Hospital of Guangzhou Medical University, Guangzhou, China; 2 Institute of Molecular and Experimental Medicine, Welsh Heart Research Institute, Cardiff University School of Medicine, Cardiff, UK

**Keywords:** Diagnosis, Stroke, Stroke mimics, ROSIER scale, FAST scale, LAPSS scale, Emergency department, China

## Abstract

**Background:**

Early identification of stroke is crucial to maximize early management benefits in emergency departments. This study aimed to develop and validate a new stroke recognition instrument for differentiating acute stroke from stroke mimics in an emergency setting.

**Methods:**

A prospective observational cohort study among suspected stroke patients presenting to Emergency Department in the Second Affiliated Hospital of Guangzhou Medical University was conducted from May 2012 to March 2013. The symptoms and signs of suspected stroke patients were collected. Logistic regression analysis was used to identify the factors associated with acute stroke. The symptoms and signs closely associated with acute stroke were selected to develop the new stroke scale, Guangzhou Stroke Scale (GZSS). The diagnostic value of GZSS was then compared with ROSIER, FAST and LAPSS. The primary outcome was confirmed stroke by CT within 24 h.

**Results:**

Four hundred and sixteen suspected stroke patients (247 ischemia, 107 hemorrhage, 4 transient ischemic attack, 58 non-stroke) were assessed. A new stroke scale, GZSS (total score from −1 to 8.5), was developed and consisted of nine parameters: vertigo (−1), GCS ≤ 8 (+2), facial paralysis (+1), asymmetric arm weakness (+1), asymmetric leg weakness (+1), speech disturbance (+0.5), visual field defect (+1), systolic blood pressure ≥145 mmHg (+1) and diastolic blood pressure ≥95 mmHg (+1). Among the four scales, the discriminatory value (C-statistic) of GZSS was the best (AUC: 0.871 (*p* < 0.001) when compared to ROSIER (0.772), LAPSS (0.722) and FAST (0.699). At an optimal cut-off score of >1.5 on a scale from −1 to 8.5, the sensitivity and specificity of GZSS were 83.2 and 74.1 %, whilst the sensitivities and specificities of ROSIER were 77.7 and 70.7 %, FAST were 76.0 and 63.8 %, LAPSS were 56.4 and 87.9 %.

**Conclusion:**

GZSS had better sensitivity than existing stroke scales in Chinese patients with suspected stroke. Further studies should be conducted to confirm its effectiveness in the initial differentiation of acute stroke from stroke mimics.

## Background

Stroke is one of the most common acute and severe diseases presenting to an emergency department (ED) [1]. The early assessment and management of stroke patients should reduce morbidity and mortality [1]. The use of a stroke screening tool to identify the symptoms and signs of suspected stroke and TIA increases diagnostic accuracy of medical staff in pre-hospital and ED [1]. The widely recommended stroke scales in the western world include the Recognition of Stroke in the Emergency Room scale (ROSIER), the Face Arm Speech Test (FAST) and the Los Angeles Prehospital Stroke Screen (LAPSS). ROSIER is a seven-item ranging score from −2 to +5 that includes the clinical history (loss of consciousness, convulsive fit) and neurological signs (face, arm, or leg weakness, speech disturbance, visual field defect). FAST contains three key elements including facial weakness, arm weakness, and speech disturbance. LAPSS consists of four history items, a blood glucose measure, and three examination items designed to detect unilateral motor weakness [2–5]. However, our previous study demonstrated these three stroke scales were not effective for differentiating stroke from stroke mimics in Chinese settings [6, 7]. The reasons may be related to the difference in factors affecting the incidence of stroke subtypes and stroke mimic in different ethnic populations [8]. Therefore, it is necessary to develop a stroke scale suitable to a Chinese emergency setting.

The aims of our study were firstly to identify factors that predict stroke, secondly to develop a new stroke scale in our emergency setting, and thirdly to compare the diagnostic value of the new stroke scale with ROSIER, FAST and LAPSS.

## Methods

### Study design

A prospective observational study of patients with suspected stroke was conducted from May 2012 to March 2013. Ethical approval was obtained from the Clinical Research Ethics Committee of the 2nd Affiliated Hospital of Guangzhou Medical University. Written consents were also obtained from all patients or the closest available relatives. Patients were informed that they might withdraw from the study at any time.

### Study setting

This study was conducted in the emergency department of the second Affiliated Hospital of Guangzhou Medical University (AHGZMU), which serves a population of approximately 1.56 million people in the Hai Zhu district, Guangzhou. It is an academic hospital with 1500 beds affiliated with the Guangzhou Medical University. The ED receives more than 150,000 new patients per annum and serves a local population of approximately 1,550,000 people.

### Inclusion and exclusion criteria

Suspected stroke patients ≥18 years old presenting to the ED with symptoms or signs within 7 days were recruited. Patients were excluded if they were <18 years old, had traumatic brain injury, subarachnoid hemorrhage, or unknown diagnoses.

### Measurements and data collection

Demographic data, clinical manifestations, risk factors, medical history information and the assessment of ROSIER, FAST and LAPSS were collected [2, 3]. The final diagnosis was made by a specialist stroke physician after assessment and review of clinical symptoms and brain imaging findings (CT or MR). All the patients were divided into stroke or non-stroke groups based on the final diagnosis. Glasgow Coma Scale (GCS) was used to assess the severity of coma (the motor score was applied to the non-affected limb) [9]. All the patients’ scores in this study were assessed by an emergency doctor who has obtained the certification of National Institute of Health Stroke Scale.

### Definitions

Stroke was defined as a focal or global neurological deficit with symptoms lasting for 24 h or resulting in death before 24 h, which was thought to be due to a vascular cause after investigation [3]. TIA was defined as clinical syndromes characterized by an acute loss of focal cerebral or monocular function with symptoms lasting less than 24 h and thought to be caused by in adequate blood supply as a result of thrombosis or embolism [3]. Vertigo is defined as the illusion of movement in space [10].

### Statistical analyses

Categorical variables were compared using Chi-square analysis, whilst continuous variables were compared using independent t-tests. Univariate analysis was initially used on all variables, and results were presented as ORs with 95 % CIs. Variables that were identified as significant from the univariate analysis (*p* < 0.05) were entered into logistic regression models to identify independent factors for differentiation of stroke from stroke mimics. The backward stepwise regression analyses were used to construct the models. Significant predictive variables generated in the first model were selected for the final model. The receiver operating characteristics (ROC) curve analysis was utilized to determine the optimal cut-off value of GZSS for discriminating between patients with stroke and stroke mimic. Diagnostic performances of the new stroke scale, ROSIER, FAST and LAPSS were also compared using ROC analysis. The sensitivities, specificities, positive and negative predictive values (PPV and NPV), positive and negative likelihood ratios (LR+ and LR-), and diagnostic accuracy were calculated. Statistical significance was set at *p* < 0.05. All analyses were performed using SPSS v17.0 (SPSS Inc, IL, USA) and Medcalc v9.5 (MedCalc Software, Mariakerke, Belgium).

## Results

### Patient characteristics

**Fig. 1:**
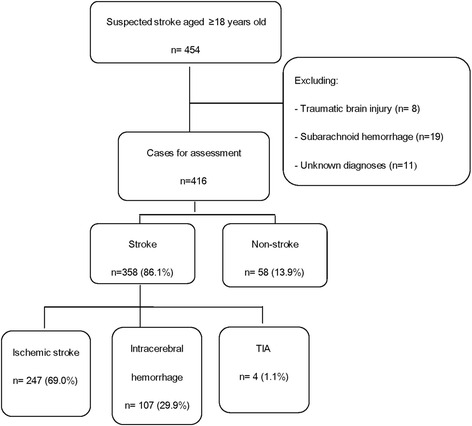
Flowchart of stroke patient recruitment

Four hundred and sixteen patients were assessed between May 2012 and March 2013. There were 358 (86.1 %) stroke cases, including 247 (69.0 %) ischemic stroke, 107 (29.9 %) intracerebral hemorrhage, 4 (1.1 %) TIA, and 58 (13.9 %) non-stroke cases (Fig. 1). Compared with non-stroke group, patients with stroke had higher systolic blood pressure (SBP), diastolic blood pressure (DBP) and incidence of several symptoms and signs including asymmetric facial weakness, asymmetric arm weakness, asymmetric leg weakness, speech disturbance, visual field defect, gaze palsy, loss of consciousness or syncope and pathologic reflex (Table 1). However, non-stroke patients had higher incidence of vertigo, nausea and vomiting. The most common stroke mimics were cervical spondylosis, seizure, peripheral vertigo, which together composed 68.9 % of non-stroke cases (Table 2). Among the 61 cases of stroke patients with GCS ≤8, there were 47 patients with intracerebral hemorrhage (77 %) and 14 patients with ischemia stroke (23 %), including 8 cases with total anterior circulation stroke and middle cerebral artery occlusion, 3 cases with partial anterior circulation stroke and stroke past history, 3 cases with posterior circulation stroke located in brain stem (Table 3).

**Table 1:** Baseline demographic characteristics of stroke and non-stroke patients (*n* = 416)

Variables	Stroke *n* = 358	Non-stroke *n* = 58	*P*
Age	69.2 ± 13.8	70.6 ± 11.4	0.397
Male, *n* (%)	210 (58.7)	37 (63.8)	0.460
SBP, mmHg	168.8 ± 31.9	147.8 ± 27.6	<0.001*
DBP, mmHg	91.8 ± 20.7	80.8 ± 14.3	<0.001*
bpm	80.9 ± 18.1	80.0 ± 13.3	0.742
Medical history, *n* (%)			
Smoker	146 (40.8)	20 (34.5)	0.339
Hypertension	202 (56.4)	34 (58.6)	0.992
Diabetes	58 (16.2)	14 (24.1)	0.157
Hyperlipidemia	40 (11.2)	9 (15.5)	0.407
Ischemic heart disease	36 (10.1)	8 (13.8)	0.434
Atrial fibrillation	28 (7.8)	1 (1.7)	0.148
Symptoms & signs, *n* (%)			
Asymmetric facial weakness	181 (50.6)	8 (13.8)	<0.001*
Asymmetric arm weakness	217 (60.6)	7 (12.1)	<0.001*
Asymmetric leg weakness	228 (63.7)	9 (15.5)	<0.001*
Speech disturbance	195 (54.5)	17 (29.3)	<0.001*
Visual field defect	68 (19.0)	1 (1.7)	0.001*
gaze palsy	115 (32.1)	5 (8.6)	<0.001*
Sensory deficits	78 (21.8)	8 (13.8)	0.163
Loss of consciousness or syncope	119 (33.2)	10 (17.2)	0.015*
Seizure activity	21 (5.9)	6 (10.3)	0.319
Pathologic reflex	102 (28.5)	8 (13.8)	0.019*
Meningeal irritation	22 (6.1)	0 (0)	0.102
Vertigo	26 (7.3)	19 (32.8)	<0.001*
Headache	25 (7.0)	3 (5.2)	0.816
Nausea	59 (16.5)	26 (44.8)	<0.001*
Vomiting	51 (14.2)	23 (39.7)	<0.001*
Score			
GCS > 8, *n* (%)	297 (83)	56 (96.6)	0.007*
GCS ≤ 8, *n* (%)	61 (17)	2 (3.4)	0.007*
GCS, median (IQR)	15 (12–15)	15 (15–15)	<0.001*

*Statistically significant difference was observed between two groups

**Table 2:** Diagnoses of stroke and non-stroke patients (*n* = 416)

Diagnoses	*N* (%)
Stroke classification	358 (100.0)
Total anterior circulation stroke	41 (11.5)
Partial anterior circulation stroke	97 (27.1)
Lacunar stroke	88 (24.6)
Posterior circulation stroke	21 (5.7)
Intracerebral Hemorrhage	107 (29.9)
Transient Ischemic attack	4 (1.1)
Non-stroke	58 (100.0)
cervical spondylosis	28 (48.3)
Seizure	6 (10.3)
Peripheral vertigo	6 (10.3)
Parkinson’s disease	2 (3.5)
Vasovagal syncope	2 (3.5)
Other^a^	14 (24.1)

^a^Other diagnoses: hyponatremia and hypokalemia (*n* = 1), hypoglycemic coma (*n* = 1), alcoholic cirrhosis (*n* = 1), overdose of clozapine (*n* = 1), chronic obstructive pulmonary disease (*n* = 1), cerebral arteriosclerosis (*n* = 1), Hashimoto’s encephalopathy (*n* = 1), vascular headache (*n* = 1), medulla oblongata and cervical vertebrae (C1-C2) focus (*n* = 1), scrub typhus (*n* = 1), central nervous system infection (*n* = 1), periodic paralysis (*n* = 1), facial neuritis (*n* = 1), left middle cerebral artery (M1) following stenting (*n* = 1)

**Table 3:** The subtype of stroke patients with GCS ≤ 8

Diagnoses	*N* (%)
Intracerebral Hemorrhage	47 (77.0)
Ischemia stroke	14 (23.0)
Total anterior circulation stroke	8
Partial anterior circulation stroke	3
Posterior circulation stroke	3
Total	61 (100.0)

### Development of the new stroke scale

Logistic regression analysis of clinical symptoms and signs for stroke and non-stroke patients are shown in Table 4. GCS ≤8 was recognized as the highest prevalence, following by visual field defect, asymmetric arm weakness, asymmetric leg weakness, SBP ≥145 mmHg, DBP ≥95 mmHg, speech disturbance and vertigo. As shown in Table 5, the items of the new stroke scale with total score from −1 to 8.5 included vertigo (−1), GCS ≤ 8 (+2), Asymmetric facial weakness (+1), asymmetric arm weakness (+1), asymmetric leg weakness (+1), speech disturbance (+0.5), visual field defect (+1), SBP ≥145 mmHg (+1), DBP ≥95 mmHg (+1). We developed the new scale based on *P* value of Multiple Logistic regression and odds ratio (Tables 4 and 5).

**Table 4:** Factors associated with stroke using logistic regression analysis

Variables		Univariate regression		Multivariate regression	
	*N* (%)	OR (95 % CI)	*P*	OR (95 % CI)	*P*
Female	169 (40.6)	1			
Male	247 (59.4)	0.81 (0.45–1.43)	0.461		
Age		0.99 (0.97–1.01)	0.457		
GCS > 8	353 (84.9)	1			
GCS ≤ 8	63 (15.1)	5.75 (1.37–24.20)	0.017*	5.70 (1.26–25.80)	0.024*
Seizure activity					
NO	389 (93.5)	1			
YES	27 (6.5)	0.54 (0.21–0.54)	0.205		
Vertigo					
NO	371 (89.1)	1			
YES	45 (10.8)	0.16 (0.08–0.32)	<0.001*	0.34 (0.15–0.74)	0.007*
Asymmetric facial weakness					
NO	227 (54.6)	1			
YES	189 (45.4)	6.39 (2.95–13.87)	<0.001*	2.44 (0.97–6.11)	0.057
Asymmetric arm weakness					
NO	192 (46.2)	1			
YES	224 (53.8)	11.21 (4.95–25.41)	<0.001*	3.39 (1.04–11.08)	0.043*
Asymmetric leg weakness					
NO	179 (43.0)	1			
YES	237 (57.0)	9.55 (4.54–20.07)	<0.001*	2.77 (0.88–8.70)	0.081
Speech disturbance					
NO	204 (49.0)	1			
YES	212 (51.0)	2.89 (1.58–5.27)	0.001*	1.29 (0.58–2.84)	0.524
Visual field defect					
NO	347 (83.4)	1			
YES	69 (16.6)	13.37 (1.82–98.23)	0.011*	3.93 (0.48–32.30)	0.202
Gaze palsy					
NO	296 (71.2)	1			
YES	120 (28.8)	5.02 (1.95–12.89)	0.001*	0.75 (0.19–3.08)	0.693
Pathologic reflex					
NO	306 (73.6)	1			
YES	110 (26.4)	2.49 (1.14–5.44)	0.022*	0.73 (0.28–1.91)	0.516
SBP ≥ 145 mmHg					
NO	111 (26.7)	1			
YES	305 (73.3)	3.99 (2.25–7.07)	<0.001*	2.55 (1.25–5.24)	0.011*
DBP ≥ 95 mmHg					
NO	270 (64.9)	1			
YES	146 (35.1)	4.62 (2.04–10.48)	<0.001*	2.29 (0.87–6.03)	0.093

*Statistically significant (*p* <0.05)

**Table 5:** Clinical signs and symptoms for development of the new stroke scale

Variables	Score	*β*	OR (95 % CI)	*P* value
Vertigo	−1	−1.09	0.34 (0.15–0.74)	0.007*
GCS ≤ 8	2	1.74	5.70 (1.26–25.80)	0.024*
Asymmetric facial weakness	1	0.89	2.44 (0.97–6.11)	0.057
Asymmetric arm weakness	1	1.22	3.39 (1.04–11.08)	0.043*
Asymmetric leg weakness	1	1.02	2.77 (0.88–8.70)	0.081
Speech disturbance	0.5	0.26	1.29 (0.58–2.84)	0.524
Visual field defect	1	1.37	3.93 (0.48–32.30)	0.202
SBP ≥ 145 mmHg	1	0.94	2.55 (1.25–5.24)	0.011*
DBP ≥ 95 mmHg	1	0.83	2.29 (0.87–6.03)	0.093

*P < 0.05

Stroke is likely if total scores are >1.5

### Diagnostic performances of ROSIER, FAST, LAPSS and GZSS

**Fig. 2:**
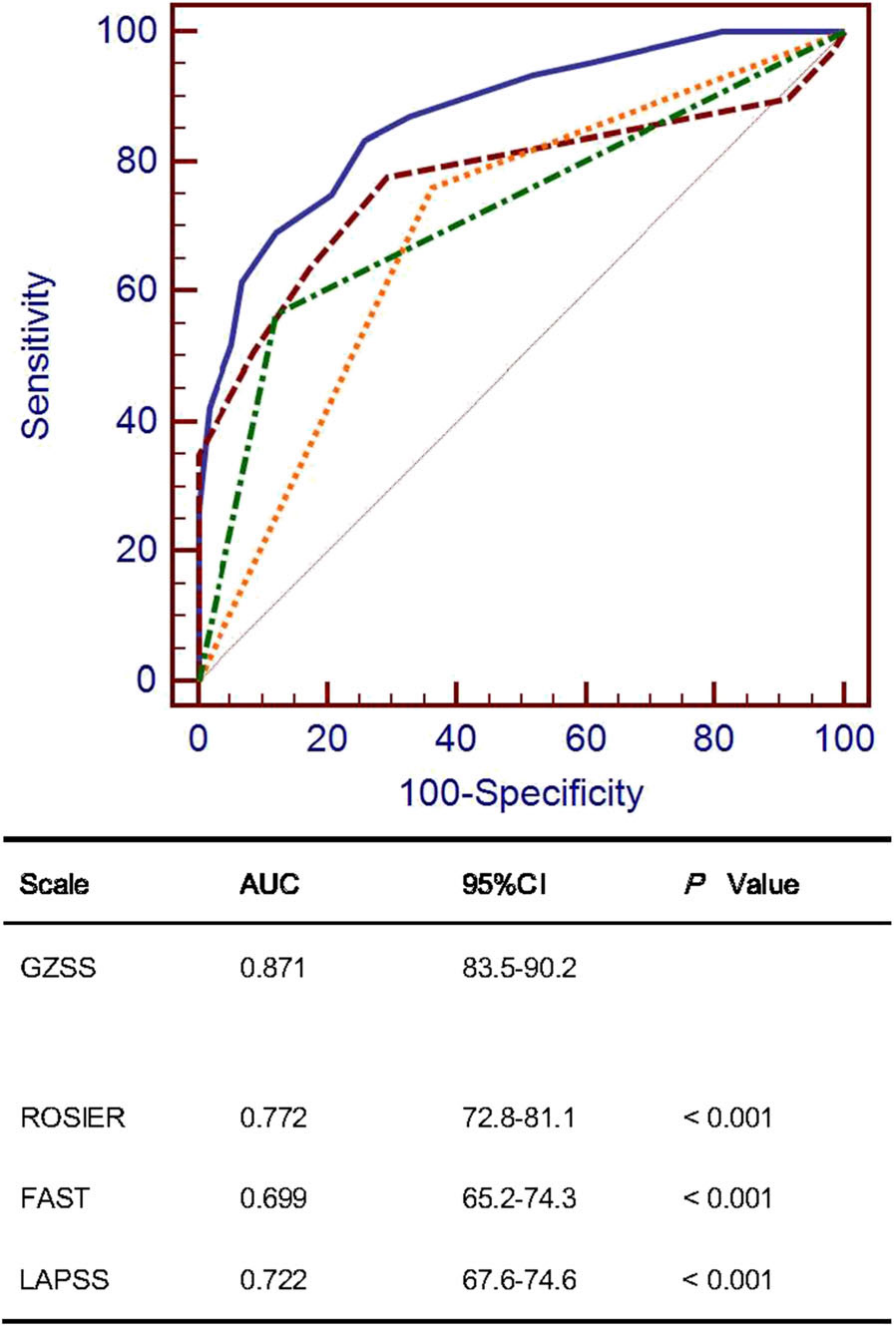
Receiver operating characteristic ROC curves of GZSS, ROSIER, FAST and LAPSS in differentiation of stroke and non-stroke patients

The nine-item scoring system for GZSS was constructed. Comparison of GZSS to ROSIER, FAST and LAPSS was undertaken using ROC analysis (Fig. 2). The area under curve (AUC) of GZSS was 0.871 (95 % CI 83.5–90.2), whilst the AUC of ROSIER was 0.772 (95% CI 72.8–81.1), LAPSS was 0.722 (95 % CI 67.6–74.6) and FAST was 0.699 (95 % CI 65.2–74.3). By pairwise comparison of the AUC of the four scales (adjusted α = 0.008), the comparison was statistically significant (*p* < 0.001). Among the four scales, the diagnostic value of GZSS was the best (Fig. 2).

The optimal cut-off score of GZSS was determined to be a total score of 1.5. At this cut-off score, the corresponding sensitivity and specificity were 83.2 % (95 % CI 79.0–87.0) and 74.1 % (95 % CI 61.0–84.7). The sensitivity and specificity of ROSIER scale were 77.7 % (95 % CI 73.0–81.9) and 70.7 % (95 % CI 57.3–81.9) when the optimal cut-off score was 0. The sensitivity and specificity of FAST scale were 76.0 % (95 % CI 71.2–80.3) and 63.8 % (95 % CI 50.1–76.0) when the optimal cut-off score was 0. The sensitivity and specificity of LAPSS scale were 56.4 % (95 % CI 51.1–61.6) and 87.9 % (95 % CI 76.7–95.0) when the optimal cut-off score was 0. The sensitivity of GZSS was the best and was significantly different from the sensitivities of ROSIER scale (*p* = 0.031), FAST scale (*p* = 0.004) and LAPSS scale (*p* < 0.001) (Table 6).

**Table 6:** Diagnostic performance of ROSIER, FAST, LAPSS and GZSS

Scale	Optimal cut-off	Sensitivity (95 % CI)	*P*	Specificity (95 % CI)	*P*	PLR (95 % CI)	NLR (95 % CI)	PPV (95 % CI)	NPV (95 % CI)	Diagnostic accuracy (%)
ROSIER	0	77.7 (73.0–81.9)	0.031	70.7 (57.3–81.9)	0.754	2.65 (1.8–4.0)	0.32 (0.2–0.4)	94.2 (90.9–96.6)	33.9 (25.5–43.1)	76.68
FAST	0	76.0 (71.2–80.3)	0.004	63.8 (50.1–76.0)	0.210	2.1 (1.5–3.0)	0.38 (0.3–0.5)	92.8 (89.2–95.5)	30.1 (22.1–39.0)	74.28
LAPSS	0	56.4 (51.1–61.6)	<0.001	87.9 (76.7–95.0)	0.008	4.68 (2.3–9.4)	0.50 (0.4–0.6)	96.7 (93.2–98.6)	24.6 (18.9–31.1)	60.82
GZSS	1.5	83.2 (79.0–87.0)	Reference	74.1 (61.0–84.7)	Reference	3.22 (2.1–5.0)	0.23 (0.2–0.3)	95.2 (92.2–97.3)	41.7 (32.1–51.9)	81.97

*ROSIER* Recognition of Stroke in the Emergency Room scale, *FAST* Face Arm Speech Test, *LAPSS* Los Angeles Pre-Hospital Stroke Screen, *GZSS* Guangzhou Stroke Scale, *PLR* positive likelihood ratio, *NLR* negative likelihood ratio, *PPV* positive predictive value, *NPV* negative predictive value

## Discussion

This was the first study to develop a new stroke scale in China. In recent years, the incidence of stroke is still rising year by year around the world and its high morbidity can cause serious social and family burden [11–14]. Early recognition and timely treatment of patients with acute stroke by emergency physicians are critical and improve the prognosis of stroke patients [15–22]. Stroke screening scales were recommended by guidelines in pre-hospital and emergency room for rapid triage of suspected patients [1, 3, 23–25].

In this study, we found that there were significant differences in the distribution of stroke subtypes between the patients in our study and the Western patients in the studies using other scales. There was a higher proportion of intracerebral haemorrhage in our study compared with the other Western studies [26–29]. Also, there were less ischaemic stroke (69 versus 76 %) and TIA (1.1 versus 10 %), but more haemorrhagic stroke (29.9 versus 14 %) in our study than in the ROSIER study [2]. These differences in subtype patterns are postulated to be due to differences in genetic, clinical, environmental and lifestyle factors [8, 30, 31]. There were also differences in the proportion of stroke mimics between this study and the other Western studies, Seizures (10.3 %) and syncope (17.2 %) in Guangzhou were less common than in the UK (47 %) [2]. Another UK study found that primary headache disorders and seizures comprised up to 27 % of stroke mimics [32], whilst in Greece [33] the principal stroke mimics were aphasic disturbances (27.3 %) and dizziness or fainting (27.3 %).

We analyzed different clinical features of stroke in our ED and thus developed a new stroke recognition instrument (score range: −1 to 8.5), which consisted of nine items including vertigo (−1), GCS ≤ 8 (+2), facial paralysis (+1), asymmetric upper limb paralysis (+1), asymmetric lower limb paralysis (+1), speech disorders (+0.5), visual field defect (+1), SBP ≥145 mmHg (+1), DBP ≥95 mmHg (+1). The new stroke scale showed good discriminative value in our ED.

In GZSS, five recognition items were the same as in ROSIER scale, FAST score and LAPSS. These items with different odds ratios were included in the new stroke scale, such as asymmetric facial weakness (OR: 6.39), asymmetric arm weakness (OR: 11.21), asymmetric leg weakness (OR: 9.55), speech disturbance (OR: 2.89), visual field defect (OR: 13.37). We assigned the corresponding score to each item of the new scale based on the logistic regression coefficients.

There were some new items added in GZSS based on the analysis. In our previous study, we found that the level of consciousness of patients may affect the diagnostic value of the stroke screening scales [2, 6, 7]. Therefore, we assessed the diagnostic value of GCS in patients with suspected stroke. We found that GCS equal or less than 8 points (OR: 5.75) was associated with the diagnosis of stroke. Stroke patients often have disturbance of consciousness and are unable to cooperate in medical examinations. It would be difficult for coma patients to carry out some of the physical examinations, such as paralysis and speech disorders. In this case, most of the stroke screening scales developed in western countries, such as FAST and LAPSS, cannot be applied to stroke patients with loss of consciousness. Also, if stroke patients present with loss of consciousness and do not have paralysis and speech disorders, the total score of ROSIER scale is equal to or less than 0 and thus means stroke is not likely to occur. This would easily lead to false negative. Therefore, we included GCS in GZSS to compensate the deficiency of the other stroke scales.

Vertigo was another new item in GZSS. Our study suggested vertigo occurred more often in Chinese non-stroke population than Western population. In the western population, the proportion of stroke and non-stroke patients with vertigo were similar (6 and 5 %, respectively) [2]. However, 58.6 % of patients had cervical spondylosis and peripheral vertigo in this study. By logistic regression analysis, the regression coefficient βof vertigo was negative, which suggested that vertigo was a differential symptom between stroke and non-stroke patients.

In addition, our new scale included SBP and DBP. We used ROC analysis to determine the optimal cut-off values of SBP (≥145 mmHg) and DBP (≥95 mmHg). By logistic regression analysis, we found that the OR of SBP and DBP were2.55 and 2.29, respectively. When acute stroke occurs, SBP and DBP are higher than usual. Blood pressure of more than 80 % of patients increased within 24 to 48 h after the onset of cerebral ischemic, and declined gradually in a few days or several weeks. One of the reasons might be due to the regulation disorder of cerebral blood flow in ischemic penumbra [34, 35]. Patients with hemorrhagic stroke experienced increased intracranial pressure, and thus the blood pressure would increase to maintain the normal cerebral blood flow. In this study, the blood pressure was higher in patients with stroke than non-stroke (*p* < 0.01). Also, 392 patients (94.2 %) including 337 stroke patients (81.0 %) and 55 non-stroke patients (13.2 %) presented to ED within 24 h after symptom onset. Only 5.8 % of patients presented over 24 h after symptom onset. This indicated that the vast majority of patients with suspected stroke in our study were in acute period of cerebral apoplexy. Therefore, blood pressure also played a significant role in the diagnosis of patients with suspected stroke in the emergency department.

A comparison of the new stroke scale with the other three scales (ROSIER scale, FAST scale and LAPSS scale) was conducted in our ED. We found that the new scale had better sensitivity than the other scales. The AUC of GZSS was the largest (AUC = 0.871). At the optimal cut-off score of 1.5, GZSS gave high sensitivity and comparable specificity. It may be more effective to use GZSS to screen Chinese patients with suspected stroke in ED.

### Limitations

There were several limitations in this study. First, this study was a single-center study. Our results may not be generalizable to other parts of China, let alone elsewhere in the world. Multicenter studies with larger sample sizes are needed to explore the effectiveness of this new stroke scale. Second, using GCS in GZSS may not be appropriate in all circumstances. A patient who has had a stroke may be aphasic and hemiplegic. They may be fully conscious but only scored E4V1M6 giving them a GCS of 11/15, which is clearly misleading. It may be necessary to break down the GCS into the component parts (i.e. E4V1M6 instead of GCS 9) to get much more information. Third, it would have been stronger to have separate derivation and validation datasets rather than a single dataset. Therefore, further studies with larger sample sizes are required to validate the effectiveness of GZSS and improve its weakness.

## Conclusion

GZSS had better sensitivity than the existing stroke scales in Chinese patients with suspected stroke. Further studies are required to validate its usefulness in the initial differentiation of acute stroke from stroke mimics.

## Abbreviations

AUC: area under the ROC curve
CT: computed tomography
DWI: diffusion weighted imaging
FAST: the face arm speech test
GCS: Glasgow Coma Scale
IQR: inter quartile range
LAPSS: the Los Angeles Prehospital Stroke Screen
MRI: magnetic resonance imaging
NIHSS: National Institute of Health stroke scale
OR: odds ratio
ROC: receiver operating characteristic
ROSIER: the Recognition of Stroke in the Emergency Room scale
TIA: transient ischemic attack
